# Vendor‐provided clinical physics services are a disservice to patients and the medical physics profession

**DOI:** 10.1002/acm2.13243

**Published:** 2021-04-10

**Authors:** Dongxu Wang, Per Halvorsen, Yi Rong

**Affiliations:** ^1^ Department of Medical Physics Memorial Sloan Kettering Cancer Center Middletown NJ USA; ^2^ Department of Radiation Oncology Beth Israel Lahey Health Burlington MA USA; ^3^ Department of Radiation Oncology Mayo Clinic Arizona Phoenix AZ USA

## 1 INTRODUCTION

Vendor‐provided commercialized equipment and software are well accepted in the medical physics profession, since they are designed to free up physicists’ time, improve workflow efficiency, and provide higher accuracy and precision. There have been clear lines dividing three parties, at least in the field of radiation oncology: patients, health care providers, and medical device suppliers. Medical physicists (and dosimetrists) are part of the health care providers group. One of their main duties, as defined by IAEA (International Atomic Energy Agency), is “mainly related to the safety and performance of related equipment and computer systems”.^1^ Therefore, any financial relationship between medical physicists and medical device vendors should be clearly disclosed to avoid conflicts of interest (COI). However, in recent years, vendors of radiotherapy equipment and software are increasingly engaged in directly providing radiotherapy physics services to the clinic. This includes not only vendor‐sponsored equipment commissioning but also vendor‐employed dosimetrists conducting treatment planning (i.e., “software as a service” or “oncology as a service”) for radiation oncologists through its business subsidiaries.^2^ The vertical integration of technical services, traditionally provided separately by vendors and clinical medical physicists or dosimetrists, may lead to unavoidable conflicts of interest and thus this parallel/opposed debate on the following opinion statement: *Vendor‐provided clinical physics services are a disservice to patients and the medical physics profession*.

Parallel to this opinion is **Dongxu Wang** ^1^. Dongxu Wang received his PhD in Medical Physics from University of Wisconsin‐Madison in 2011. After graduate school, he joined the University of Iowa Hospitals and Clinics as an Assistant Professor in Medical Physics. At the University of Iowa, he studied part‐time and received his Master’s degree in Business Administration (MBA) in 2019. He is now Associate Attending Physicist at Memorial Sloan Kettering Cancer Center. Dr. Wang’s earlier expertise and focus were in proton therapy and proton imaging. Recently he became an active member of the Medical Physics Leadership Academy (MPLA), with a particular interest in using the case study method to advance the leadership and professionalism education for medical physicists.

Opposed to this opinion is **Per Halvorsen**. Mr. Halvorsen is the Chief Physicist for the Lahey division in Radiation Oncology at Beth Israel Lahey Health in suburban Boston. He received his MS degree in Radiological Medical Physics from the University of Kentucky in 1990 and was certified by the American Board of Radiology in 1995. He has been a member of the American Association of Physicists in Medicine (AAPM) for nearly 30 yr and has been an active volunteer in professional societies, chairing the AAPM Professional Council and serving on the Board of Directors. He has authored numerous peer‐reviewed manuscripts, recently as the chair of the Medical Physics Practice Guideline for Stereotactic Radiosurgery (SRS) and Stereotactic Body Radiotherapy (SBRT) and as a member of the ASTRO–ASCO–AUA Evidence‐Based Guideline for Hypo‐fractionated Prostate treatment. He is a volunteer surveyor for the American College of Radiology (ACR) and served many years on its accreditation program oversight committee. He is Deputy Editor‐in‐Chief of the JACMP, and a Fellow of the ACR and AAPM.

## 2 OPENING STATEMENT

### 2.A Dongxu Wang

Medical physicists and medical dosimetrists add clinical values to cancer care. Some of our services, such as treatment planning and special physics consults, are billable and therefore adding financial values as well. Driven by financial interests, as a corporate would rightfully do, some vendors want to provide medical physics and medical dosimetry services as an extension of their technical product. Serious concerns have already been raised regarding regulatory ambiguity and ethical dilemma with vendors’ involvement into routine clinical practice, as a disservice to patients and to our profession, and eventually to their core business.

#### 2.A.1 The regulatory ambiguity

Radiotherapy physicists, as health care providers who are independent from medical device vendors, assume the responsibility of ensuring an FDA‐cleared device is at least safe and effective. It is a key to understand that radiotherapy devices, categorized as Class II, are not subjective to extensive testing and clinical trial before FDA 510(K) clearance. Vendors certainly do not want to assume this additional liability in the first place. Any physicists who have been the early users of a newly developed radiotherapy equipment can attest to the pain and effort, despite the joy and satisfaction, in all kinds of troubleshooting for a new, advanced but complicated technology. It is important to note that not all such troubleshooting is for trivial or non‐harmful flaws; rather, some of those may be significant enough to cause harm or even death to a patient, if they had not been caught by a diligent and experienced clinical physicist.

A vendor‐employed physicist or dosimetrist may be potentially liable for medical device defects. For example, if a dosimetrist employed and trained by a treatment planning system (TPS) vendor made a mistake in dose calculation due to a software design bug in the same TPS, is that a professional error or medical device error? With “software as service” being the business concept, is there still a boundary between a medical device and professional service? We can see two extreme outcomes with this situation. The dosimetrist alone can serve as a scapegoat, to avoid hurting sales of the TPS software. Or, the regulator may scrutinize the medical device vendor as an entirety. Heightened regulation will destroy the current entrepreneurial environment where cancer‐curing technology advances fast.

#### 2.A.2 The ethical dilemma

At present, most clinical physicists are employed by hospitals and their main duties are caring for patients. Medical physicists sometimes help vendors improve their products in commissioning and troubleshooting a new device. Had the physicists been employed by the machine vendor, who do they serve or report to? Some of the common vendor practices would be otherwise stroking serious COI concerns. A significant portion of workflow improvement or troubleshooting for a new device may take place during the treatment of a real patient, since anything unexpected can happen. And this highly depends on the physicists who are responsible for testing and commissioning the device. Now if the medical physicist is an extension of the vendor, this whole troubleshooting process could be undisclosed and patients might suffer from a nonconsented clinical trial of a prematurely engineered medical device.

The prospect of medical physics being an extension of a medical device vendor’s service brings up entangled and conflicted interests. The divided professional loyalty by medical physicists is likely viewed as detriments to the best patient care. This endangers medical physics profession as medical physicists may no longer be considered the trustworthy partners of clinicians or the radiation safety gatekeepers for patients. The credibility of medical physics as an independent, board‐certified healthcare profession will be at risk. Imagine a physician practice owned by a pharmaceutical company; I will think twice before seeing a doctor there, if ever.

### 2.B Per Halvorsen

First, I should clarify that I am personally ambivalent about this topic, but the topic merits a robust debate without the distraction of focusing on any particular vendor, so I volunteered to articulate the opposing view. I am ready for the eggs.

My current employer is a not‐for‐profit regional health system, but I previously served as Vice President for Medical Physics for a national for‐profit radiation oncology service provider. In preparing for this debate, I also spoke with several physicist colleagues who are currently or were previously employed by one of the national equipment vendors. Two comments in particular stuck with me: “As a profession we’re defined by both the top and the bottom” (the latter referring to physicians in rural communities who have witnessed the effects of poor radiotherapy services) and “The whole reason we have this option is because there’s a need. Any solution needs to be weighed against the option of not addressing the need.”

As a profession, I believe we should recognize three uncomfortable truths:

1. Access to cancer care is uneven and approaches unacceptable levels in many rural areas in the United States;
2. The quality of clinical radiotherapy physics services is more variable than it should be; and
3. Clinical physicists in solo practice may fall into a “protective bubble” that does not serve patients well.

I will address each of these points, and posit that vendor‐provided clinical physics services can, when rendered in the right way, be part of the solution.

A recent study describes the disparity in access to cancer care in the United States.^3^ Approximately 60 million Americans live in areas considered rural by the United States Census Bureau,^4^ and outcomes are generally worse for this patient population.^5^ My vendor‐employed colleagues assert, and my personal experience confirms, that clinical physics coverage is considerably more variable, and more sparse overall, in such rural settings. It is not uncommon that centers rely on a medical physicist who visits the center once per month (usually outside clinical hours) to perform minimal regulatory compliance work without any opportunity for collaboration with the full clinical team to understand and improve their processes.

A recent publication in the Red Journal substantiates the second uncomfortable truth: the quality of clinical radiotherapy physics services is too variable. Kearns and colleagues reported that nearly one in five institutions were found to have errors in their TPS models, and on average, a generic reference model had better accuracy than the institution’s TPS beam model.^6^

Perhaps most uncomfortable is the risk that a solo physicist may fall into a “protective bubble.” I worked as a solo physicist many years ago, and the experience was a strong motivation for AAPM Report 80^7^ which, in turn, led to the TG‐103 report recommending peer review for physicists in solo practice.^8^ A small hospital or clinic employing a solo physicist has few points of reference for evaluating their clinical physics needs, and a friendly, helpful, and ever‐present solo physicist may develop a “walk on water” aura among the clinical and administrative colleagues. Consequently, it may be difficult for the physicist to explain their limitations with respect to commissioning new technology or commencing a new clinical service.

So, how could vendor‐provided clinical physics service be part of the solution to these concerns? First, the major vendors have a significant footprint in the radiation oncology community and may be able to pool resources (both human and technology) to serve rural clinics in a manner that no single clinic or health system could match. Second, the major vendors benefit from a large database of similar systems and employ standardized processes and reporting tools to compare every center’s systems to their national database to protect against outliers. Third, working as part of a larger team ensures that peer review is an integral component of the medical physics service. Finally, physicists working for a major vendor have access to shared tools that may not be otherwise accessible to a small center.

What about conflicts of interest? Vendor‐employed clinical physicists face an inherent conflict of interest which must be actively managed. COI can occur in any employment model,^9^ but the vendor‐employed model presents some obvious potential COI, particularly during acceptance testing and commissioning of new equipment manufactured by that vendor. Standardized reporting tools can help in this regard (so an individual physicist cannot gloss over unfavorable details), as can comparison of local data to a large aggregate data pool (to confirm equipment and local physicist performance). Finally, local clinic authority via a clear governance model can play a constructive role, such as the medical director having final say on satisfaction with the clinical physics services and having those services benchmarked against relevant AAPM reports and ACR‐AAPM Technical Standards. And last but not least, every physicist is personally responsible for living up to the Principles in AAPM Code of Ethics,^10^ regardless of their employment model.

Our vendor‐employed colleagues are competent, committed clinical physicists who care about their professional reputations. Similarly, these vendors have every incentive to safeguard their company’s reputation. In this respect, all parties are incentivized to perform to the expected professional standards. We have significant challenges with access to care and consistent quality of clinical physics services in the United States. The specific employment model is not the root cause of our most pressing challenges.

## 3 REBUTTALS

### 3.A Dongxu Wang

I appreciate Mr. Halvorsen’s insights regarding the disparities of clinical physics in rural areas. I share his wish that the vendors may help fulfill the needs for consistent and available medical physics services. Unfortunately, I do not think it will work out that way, by design or by chance, because the underlying causes for these two problems will not be addressed.

Access of medical physics services in rural area: Through my former state university appointments, I had the privilege to have served a handful of Midwest community hospitals, all being single‐Linac operations and each requiring no more than 0.6 FTE (full‐time equivalent) physicist. Indeed, medical physicists are sparser than the geographic distribution of linear accelerators themselves. I do not think a vendor can sell more physics FTE to a community practice than the current level, nor is a vendor willing to dedicate more physicists than what is worth it in the contract. More importantly, the logistical and supply–demand challenge of hiring and staffing physicists in the vast, sparsely populated rural area is almost insurmountable. As I often heard in those days, “small places are small because people don’t move there.” This challenge will not diminish just because a vendor is now providing the service, so long the service is still onsite. In fact, it can make the situation worse, now that a physicist is no longer vendor neutral. The staffing of field service engineers (FSE) from vendors is of good reference. I knew of a FSE who covered a 300‐mile area stretching along the Mississippi River, and once waited 5 h during daytime for his arrival to fix a down Linac. As bad as he felt about the long delay of timely service, unfortunately, it might just be the vendor’s good business model concerning profitability vs service level.

Consistent quality of medical physics services: I concur with Mr. Halvorsen that on vendor’s platform, data collection and quality control will be a lot easier. Consistent medical dosimetry and medical physics services should be expected. This might lead to higher quality as well, if the consistent standard is set high. However, medicine is a multifactorial endeavor; there is a general lack of direct evidence that any particular technical standard leads to discernable clinical outcome. This leaves plenty of room for interpretation for a range of acceptable technical specifications, and the possible justification for not having to achieve the best specification. This is likely to happen when profitability‐to‐effort ratio is concerned, as the business metric is much easier to establish and quantify. For example, if the vendor’s new vision is to simplify Linac commissioning by spot checking and to complete the whole process within two business days by skipping 3D water tank scanning, a vendor‐hired physicist will have to align his or her practice with the organizational direction. I do not see it possible for this vendor‐employed physicist to insist on a full range of beam data collection, occupying the company’s sparse (sounds familiar?) resource including the traveling installer, just to double check and independently confirm, as physicists are supposed to do.

I do not disagree with Mr. Halvorsen that a well‐designed governance model might mitigate some of the conflict‐of‐interest concerns with vendor‐employed clinical physicists. Yet, this practice places medical physicists as the service branch of a medical device company, not as independent practitioners in healthcare. This alters the nature of our profession as we know it. Conflict of interest sounds theoretical and manageable until one’s paycheck is at stake.

### 3.B Per Halvorsen

Dr. Wang argues that vendor‐provided clinical physics services are subject to regulatory ambiguity — that professional services will be conflated with equipment performance, potentially leading to increased regulation which “will destroy the current entrepreneurial environment where cancer‐curing technology advances fast.” I do not find this argument compelling. First, the regulators are quite adept at distinguishing between equipment safety and professional practice and maintain a firewall against unduly imposing on the practice of medicine. Second, the equipment vendors are keenly aware of the regulatory and legal environments (likely more so than most hospital‐employed clinical physicists) and would take prudent measures to protect their business from such risks. Practicing in accordance with nationally recognized practice standards and recommendations is arguably one of the most effective approaches in that regard.

I share Dr. Wang’s concerns regarding the ethical dilemma of loyalty to the patient and one’s employer. This is a “healthy tension” faced by all clinical physicists regardless of their employment model, although I agree it may be felt more acutely when the clinical physicist is employed by the manufacturer of the equipment used in the clinic. The AAPM Code of Ethics^10^ clearly states that our primary obligation is to the safety and welfare of the patient. Principle I states “Members must hold as paramount the best interests of the patient under all circumstances”, and Principle X states “Members are professionally responsible and accountable for their practice, attitudes, and actions, including inactions and omissions.” Dr. Wang mentioned that early troubleshooting for a new device often involves clinical physicists who are commissioning the equipment for use in their clinics. If such clinical physicists are employed by the equipment manufacturer, their paramount priority remains the best interests of the patient — and they may in fact be able to more effectively pursue troubleshooting and improvement of such new devices as they will have direct access to their colleagues on the product development team.

As I stated earlier, the quality of clinical radiotherapy physics services is demonstrably more variable than it should be.^6^ That may be partly due to the prevalence of isolated practices without the benefit of working with a larger team of physicists who can share expertise, tools, and information. There are many models of group practice, of course, health systems and consulting groups being two examples. There is no inherent reason why a vendor‐owned physics group practice would be an inferior model for clinical physics services.

To address the concern about potential COI with vendor‐provided clinical physics services, the hospital contracting for such services should insist on local clinic authority via a clear governance model and on having those services benchmarked against relevant AAPM reports and ACR–AAPM technical standards.

The uncomfortable truth is that, despite all our wealth as a society, we have significant challenges with access to care and consistent quality of clinical physics services in the United States. I do not believe that the specific employment model represents one of our most pressing challenges.

## Note

^1^ Views and opinions expressed here are this author’s alone, and do not represent those of Memorial Sloan Kettering Cancer Center. Dongxu Wang discloses the following Conflict of Interest: the author is a beneficiary of an intellectual property licensed to Ion Beam Applications, S.A.
